# Wafer-level integration of self-aligned high aspect ratio silicon 3D structures using the MACE method with Au, Pd, Pt, Cu, and Ir

**DOI:** 10.3762/bjnano.11.128

**Published:** 2020-09-23

**Authors:** Mathias Franz, Romy Junghans, Paul Schmitt, Adriana Szeghalmi, Stefan E Schulz

**Affiliations:** 1 Nano Device Technologies, Fraunhofer Institute for Electronic Nano Systems ENAS, Technologie-Campus 3, 09126 Chemnitz, Germany; 2 Fraunhofer Institute for Applied Optics and Precision Engineering IOF, Center of Excellence in Photonics, Albert-Einstein-Straße 7, 07745 Jena, Germany; 3 Institute of Applied Physics, Friedrich-Schiller-University Jena, Albert-Einstein-Straße 15, 07745 Jena, Germany; 4 Center for Microtechnologies, Chemnitz University of Technology, Straße der Nationen 62, 09111 Chemnitz, Germany

**Keywords:** black silicon, bottom-up, metal-assisted chemical etching (MACE), nanowires, wafer-level integration

## Abstract

The wafer-level integration of high aspect ratio silicon nanostructures is an essential part of the fabrication of nanodevices. Metal-assisted chemical etching (MACE) is a promising low-cost and high-volume technique for the generation of vertically aligned silicon nanowires. Noble metal nanoparticles were used to locally etch the silicon substrate. This work demonstrates a bottom-up self-assembly approach for noble metal nanoparticle formation and the subsequent silicon wet etching. The macroscopic wafer patterning has been done by using a poly(methyl methacrylate) masking layer. Different metals (Au, Pt, Pd, Cu, and Ir) were investigated to derive a set of technologies as platform for specific applications. Especially, the shape of the 3D structures and the resulting reflectance have been investigated. The Si nanostructures fabricated using Au nanoparticles show a perfect light absorption with a reflectance below 0.3%. The demonstrated technology can be integrated into common fabrication processes for microelectromechanical systems.

## Introduction

Silicon nanostructures significantly boost the performance of modern sensors, energy storage devices, or energy harvesters and have become essential in their development. These structures can either be well-defined regular structures fabricated in a top-down process, or self-assembled random structures from bottom-up processes. These random structures are typically vertically aligned nanowires, also called nanorods.

Silicon nanowire arrays can be designed to have a low reflectance of about 1% in a broad spectral range, depending on their geometry. These silicon structures exhibit efficient light trapping because photons are scattered within the nanostructures and are optimally delivered to active regions of the device [1]. An application for this effect is to fabricate antireflective surfaces, which has been extensively studied not only for semiconductors, but also for glass and polymer surfaces [2–3]. This property is widely used to fabricate high-efficiency solar cells [4–5]. These nanowire-based solar cells show a higher short circuit current and a higher quantum efficiency than planar cells [6]. Another energy conversion application for nanowires is a thermoelectric harvester. The one-dimensional structures reduce heat transport and improve the efficiency of the thermoelectric generator [7].

Silicon nanowire arrays are also an emerging anode material for integrated lithium-ion batteries. They have a ten times higher theoretical capacity than graphite and can be used for cells with high energy density. However, these features cannot be achieved with dense silicon, i.e., a nanoporous silicon anode is required for a successful integration [8]. Also, integrated capacitors can benefit from the increased surface of nanowires with high aspect ratio. In combination with atomic layer deposition, one can fabricate integrated metal–insulator–semiconductor or metal–insulator–metal capacitors with a high effective area on a small footprint.

The high surface area of a silicon nanowire array can be used to fabricate ion-sensitive field-effect transistors (ISFETs) with a high signal-to-noise ratio. An ISFET is a pH sensing platform and can be adapted to detect biomolecules [9]. Silicon nanowires are used as template for cancer sensors. The nanowires are implemented as gate in integrated sensing FETs [10–11]. A wide range of chemical sensors and biosensors benefit from porous silicon structures [12].

All these presented applications rely on 3D silicon nanostructures with a high aspect ratio. For an integration into a silicon-based microsystem it is essential to fabricate the silicon nanostructures locally on a pre-defined area on the system die. This demands wafer-level processes for the whole process chain. Ideally, this fabrication uses cost-efficient subprocesses and omits expensive processes such as nanopatterning with high-resolution lithography.

One low-cost method for the fabrication of such high aspect ratio templates and structures is metal-assisted chemical etching (MACE). This process uses a noble metal catalyst layer for the wet etching of silicon. The MACE process has been extensively studied over the last decade [13–16]. In theory, the process works with a wide range of noble metals. The main focus in research has been set on the noble metals gold (Au) and silver (Ag) [2,17–19]. Other studies also investigated palladium (Pd), platinum (Pt), and to a lesser extent even copper (Cu) [20–22]. However, there is no study analysing iridium (Ir) as catalytic metal. According to its standard reduction potential of 1.16 V, it is comparable to Pt (1.18 V) and Pd (0.95 V) and should work comparably as catalyst [23].

Chartier et al. [13] reported the etching mechanisms of the MACE process. The cathodic reaction is the reduction of H_2_O_2_ at the noble metal interface within an acidic solution. This reduction transfers an electron to the H^+^ ion and produces a hole (an electron vacancy) h^+^:

[1]
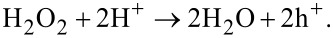


The electron vacancies play an important role within the anodic reactions at the silicon surface. The combined reaction can be written as:

[2] $$\text{Si}+6\text{HF}+n{\text{h}}^{+}\to {\text{H}}_{2}{\text{SiF}}_{6}+n{\text{H}}^{+}+\frac{4-n}{2}{\text{H}}_{2}↑.$$

This reaction summarises two etching mechanisms related to *n*, the number of consumed holes h^+^ per etched Si atom. The first mechanism is correlated to *n* = 4. The silicon will be oxidised to the intermediate state SiO_2_, which is etched by HF. In this case no H_2_ is formed. Due to the high number of involved holes, this etching mechanism is strongly correlated to the interface of silicon and the noble metal. This regime will lead to a straight silicon etching profile underneath the noble metal [13].

The second mechanism involves two holes (*n* = 2). Per etched Si atom one H_2_ molecule is formed. This etching mechanism is not limited to the metal–silicon interface and occurs in a wider area around the noble metal. This etching mechanism mainly results in nanoporous sponge-like structures [13].

Chartier et al. also investigated the influence of the relative HF concentration in comparison to the H_2_O_2_ concentration. For high relative HF concentrations, the etching rate is mainly influenced by the H_2_O_2_ concentration. Within this regime they reported pore formation as the dominant mechanism [13].

This work presents the integration of the MACE process at the wafer level using several metal nanoparticles as catalysts for the reduction process (Equation 1). The aim is to generate a high aspect ratio template of silicon. This template has to be fully integrable into a common MEMS process flow. This means that the etched silicon structures have to be fabricated within a pre-defined area of the die with common semiconductor fabrication processes. These nanostructure templates are the basis for a further processing towards integrated functional systems.

## Results and Discussion

The fabrication of the wafer-level integrated nanostructure templates is divided into two main parts, i.e., the formation of the noble metal nanoparticles, and the subsequent silicon wet etching process. The subsidiary process steps are standard semiconductor processes and therefore not analysed and discussed in detail.

### Particle formation

The surface of the deposited noble metal films has been analysed before and after the thermal annealing using a scanning electron microscope (SEM). The generated particles have been analysed using “ImageJ” [24] with the package collection “Fiji” [25]. For the analysis, we used images with a magnification of 100.000×, where each pixel has a size of 1.1 nm/pixel. The analysed area of each image has a size of 1024 × 703 pixel^2^, which represents 1126 × 773 nm^2^ = 0.89 μm^2^.

**Figure 1:**
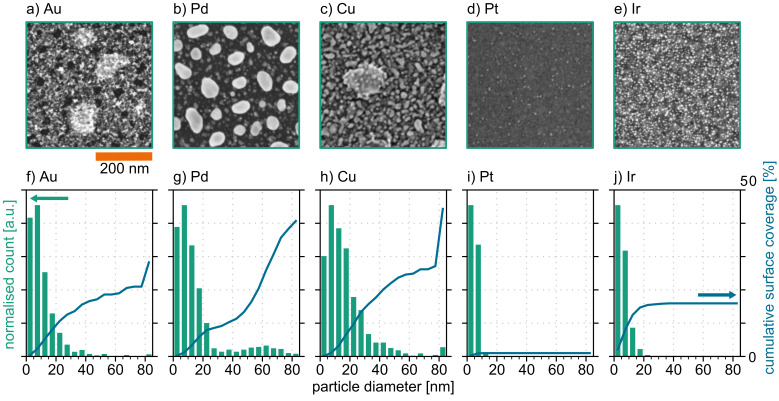
Fabricated noble metal particles. (a–e): SEM images of wafer surface; (f–j): particle size distribution (bars) and cumulative surface coverage of the particles (solid lines).

Initially, the sputtered metals formed continuous films with randomly distributed pinholes at the nanometre scale. The target film thickness was 5 nm and was adjusted using the sputtering rate. Figure 1 shows SEM images after the annealing process (Figure 1a–d) and the results of the particle distribution analysis (Figure 1f–j). Figure 1e shows the surface of the Ir sample directly after the ALD process with 45 cycles. The Ir samples were not annealed thermally. The given scale bar is representative for all SEM images within this figure. The metal films have segregated into small particles on the Si surface. For the analysis, we approximated the particles with the best fitting ellipses. The calculated diameter of each particle is derived from the mean diameter of these ellipses. The histogram plots (bar charts) in Figure 1f–j represent the normalised distribution of the particle diameters. The category width is 5 nm. The continuous line in Figure 1f–j shows the cumulative surface coverage of the given particles. The last category represents all particles with a diameter greater than 80 nm.

Within all particle classes, we could detect a high amount of ultrasmall particles with a diameter below 10 nm. For Au, Pd, and Cu, one can also observe larger particles up to a diameter of 80 nm. Here, these larger particles contribute significantly to surface coverage. The Au film covers 28% of the Si surface, while the Pd film covers more than 40% of the surface. This surface coverage comprises 1522 particles/μm^2^ at the Au wafers and 845 particles/μm^2^ at the Pd wafers, respectively. The wafers with copper show a broad particle size distribution. The surface is covered by more than 44%, which means 876 particles/μm^2^.

The Pt particles mainly have a diameter below 10 nm with 495 particles/μm^2^. The total surface coverage did not exceed 1.0%. This result implies that the Pt film was significantly thinner than the other films. The Pt deposition rate has to be re-evaluated. The particle distribution is independent of the local position on the wafer. The given SEM images were taken at the wafer centre. Control measurements on the wafer edge show a comparable distribution. This uniformity is an essential requirement, which has to be fulfilled for a successful integration at the wafer level.

**Figure 2:**
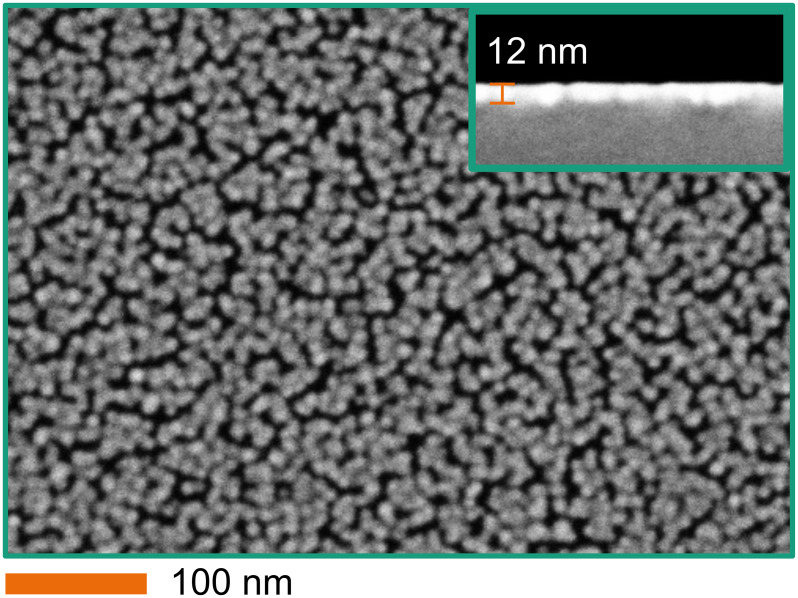
SEM surface images after 90 cycles of Ir ALD. The inset shows the corresponding cross section.

Ir (after 45 ALD cycles) has agglomerated in small (less than 15 nm) particles, which are randomly distributed on the sample surface. Here, 16% of the surface is covered with Ir particles. As the deposition was done by atomic layer deposition (ALD), one can assume that a wafer-level integration is feasible. Figure 2 shows the surface of a silicon die after 90 cycles of Ir ALD. In this deposition phase, small Ir particles are grown. The particles agglomerate and start to form a continuous film. However, there are still voids between the connected Ir clusters. The SEM cross-section measurements show that the film has an average thickness of approx. 12 nm (see inset of Figure 2).

The fabricated nanoparticles show a good compromise between process complexity and surface coverage with nanoparticles. With this bottom-up approach, we were able to cover the whole wafer surface homogeneously with nanoparticles. The particle size distribution can easily be modified by varying film thickness and annealing conditions or, correspondingly, the number of ALD cycles for the Ir particles.

### Silicon etching

The wafers with noble metal nanoparticles and a poly(methyl methacrylate) (PMMA) patterning were etched in a diluted HF/H_2_O_2_ solution for 10 min. The process was a single-wafer bath process and can be scaled up to a batch process easily. As a reference, one wafer with gold nanoparticles was etched in a HF solution (1.73 mol/L) without the oxidation agent, which leads to no significant etching results, except for isolated trenches of less than 2 nm in diameter. This result shows that the oxidation agent, here H_2_O_2_, is mandatory for the MACE process.

The wet etching with an oxidation agent is carried out with low H_2_O_2_ concentrations in the range of 10–240 mmol/L. This is within the pore formation regime according to Chartier et al. [13]. During the process, a gas formation could be observed for all samples. This phenomenon occurred solely at the uncovered areas. According to the reported etching mechanism, this outgassing is a result of the H_2_ formation during the silicon etching. This indicates that the reactions are at least partially within the second etching regime (where *n* = 2).

**Figure 3:**
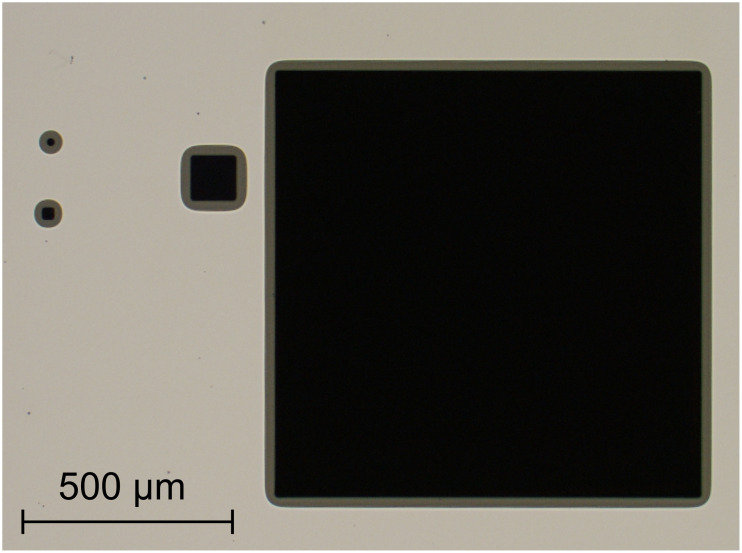
Microscope image of etched structures.

Figure 3 shows a microscope image of a wafer with Au nanoparticles after wet etching. The PMMA masking layer is still on top of the wafer. The resist slope on the structure edge is clearly visible. Although the wafer surface is entirely covered with Au nanoparticles, just the pre-defined quadratic areas are etched. The etched areas appear dark black as expected for high aspect ratio silicon nanowire arrays. This absorption phenomenom has been analysed by Fazio et al. [1]. It is mainly based on multiple light scattering at the silicon nanowires.

**Figure 4:**
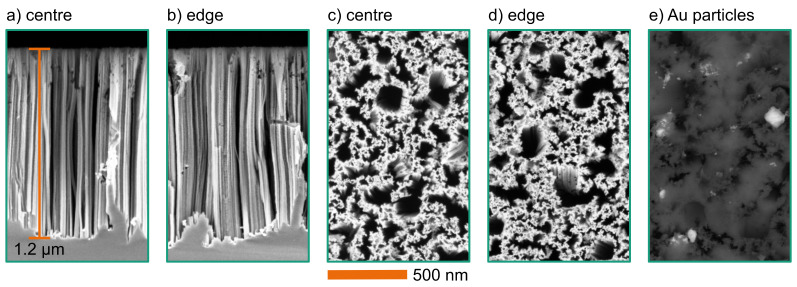
SEM images of etched structures (with 50 mmol/L H_2_O_2_ and 1.73 mol/L HF for 10 min) using Au particles. (a, b): Cross section; (c, d): top view; (e): top view from back-scattering detector with highlighted Au particles. The scale applies to all images.

Figure 4 shows SEM images of an etched wafer using Au nanoparticles as etching catalyst. This wafer has been etched with 50 mmol/L H_2_O_2_ for 10 min. The SEM images show cross sections of the wafer centre (Figure 4a), of the wafer edge (Figure 4b), and top-view images of centre (Figure 4c) and edge (Figure 4d). The cross sections of the wafer centre and the wafer edge show straight vertical structures. The average etching depth is 1.2 μm. The top-view images show that the structure consists of randomly distributed walls with trenches of various sizes. This structure comprises nanopores of a few nanometres in diameter. The observed diameter distribution of the vertical trenches corresponds to the particle size distribution of Figure 1f. This indicates that all particles contribute to the etching process. Even the sub-10 nm particles are not entirely removed from the surface during the wet etching process.

Any differences between various positions on the wafer are correlated to the random particle distribution. The exemplary SEM images from the centre and the edge show that the process does not vary significantly on the wafers. The process is quite homogeneous over the whole wafer. The straight etching profile indicates that the main etching occurred in the first etching regime (where *n* = 4).

The top-view SEM image in Figure 4e has been acquired with increased electron energy. Using a back-scattering detector with high material contrast, the Au nanoparticles at the bottom of the structures are highlighted. This image shows that the Au nanoparticles remain at the bottom of the etched structures. The catalytic nanoparticles are not consumed during the etching and have to be removed afterwards, if necessary. This has not been investigated within this work.

**Figure 5:**
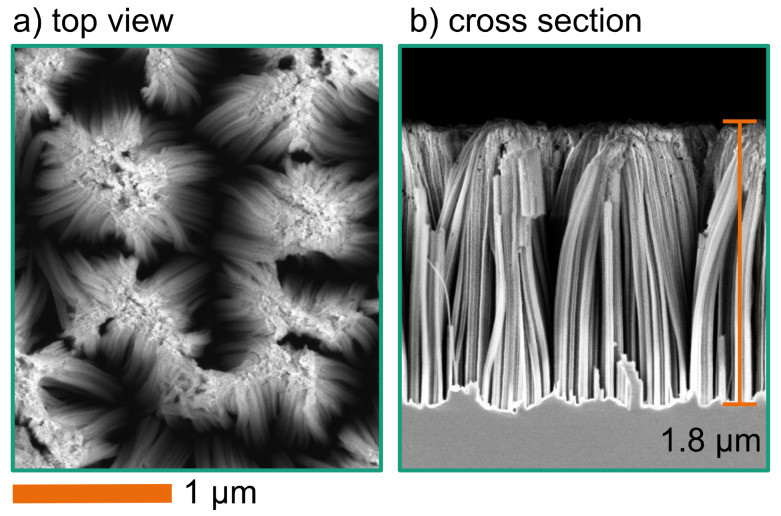
SEM images of etched structures (with 145 mmol/L H_2_O_2_ and 1.73 mol/L HF for 10 min) using Au particles showing sticking structures. (a): Top view; (b): cross section.

Up to this etching depth, the nanostructures are stable and withstand the drying process. There is no agglomeration or pattern collapse. However, with increasing etching depth, the structures become more fragile. Figure 5 shows SEM images of a wafer with Au particles after the silicon etching process. During this process, the H_2_O_2_ concentration in the etching solution was 145 mmol/L. One result of the increased H_2_O_2_ concentration is the larger etching depth of approx. 1.8 μm. Again, the etching resulted in vertical silicon walls/wires and the main etching mechanism remains the same. However, these structures stick together at the topmost point. The nanostructure bundles form during the drying process. This occurs when the capillary forces during drying are high enough to cluster neighbouring single Si wires, and the adhesion force of the bundle is larger than the elastic force [26]. This bundling effect is the main limiting factor of this integration scheme. To overcome this issue, one can use liquids with low surface tension or supercritical CO_2_ [27]. However, this would increase complexity and cost of the process significantly.

**Figure 6:**
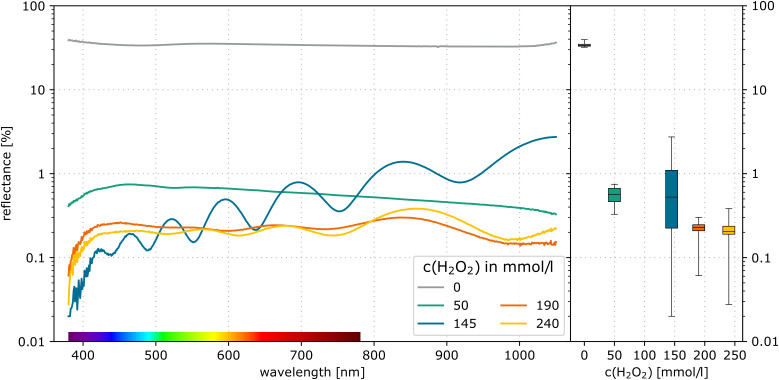
Measured reflectance of Au wafers after 10 min of etching in HF (1.73 mol/L)/H_2_O_2_ solution.

Although some wafers showed agglomerations in the SEM measurements, the etched surfaces appeared dark black. The reflection behaviour has been analysed at the centre of the large square structure with 1000 μm edge length (see Figure 3). Figure 6 shows the reflectance of the etched squares on the wafers with the Au nanoparticles. The reflectance of visible and near-infrared light (380–1050 nm) is shown on the left side of the graph. The right side summarises this range within a boxplot (see [28]). The wafer, etched without H_2_O_2_, shows a high reflectance of approximately 34%. The process with 50 mmol/L H_2_O_2_ solution shows a flat homogeneous absorption behaviour with a mean reflectance of approx. 0.56%. This result is comparable to those of the experiments of Fazio et al. [1] and Azeredo et al. [2]. Both could demonstrate a mean reflectance of less than 1% in this spectral range. This low reflectance is caused by multiple light scattering and reflection within the nanostructures [1].

The wafers etched with a higher H_2_O_2_ concentration show a lower mean reflectance. This lower reflectance is correlated to the increased etching depth with increasing H_2_O_2_ concentration. All these wafers contain nanostructures with a bundling effect at the surface. This is likely the reason for the local reflection minima and maxima. Similar results have been demonstrated and simulated by Xi et al. [29]. They reported interference effects related to the bundling of silicon pillars and correlated these effects with the moth-eye effect. The samples etched with 190 mmol/L and 240 mmol/L H_2_O_2_ showed an average reflectance of 0.22%.

**Figure 7:**
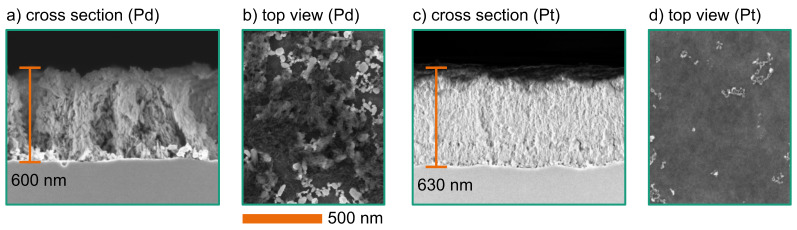
SEM images of etched structures (with 50 mmol/L H_2_O_2_ and 1.73 mol/L HF for 10 min) using Pd particles ((a): cross section; (b): top view) and Pt particles ((c): cross section; (d): top view).

Figure 7 shows the etching results for wafers covered with Pd (Figure 7a,b) or Pt (Figure 7c,d) nanoparticles. Both were etched with an H_2_O_2_ concentration of 50 mmol/L. The etching processes created nanoporous sponge-like structures, which consist of various pinholes with a diameter below 1 nm. This shows that the etching was dominated by the second etching mechanism where *n* = 2. Higher H_2_O_2_ concentrations lead to significant structural destruction, including the delamination of wide areas.

The etching process with Pd yields bundles of nanoscopic sponge structures. They are separated by larger free spaces formed by agglomerated Pd particles (Figure 7b). The mean etching depth was approx. 600 nm at the wafer centre. The wafer edge showed significant structure delamination.

In contrast to the Pd-covered wafers, the Pt-covered wafers exhibited a homogeneous sponge-like structure without a bundling effect (Figure 7c,d). The structures are approx. 630 nm deep. The Pt particles seem to remain on the surface but show a partial agglomeration. This effect can be seen in Figure 7d. Despite the Pt particles, the surface of the etched structures remains smooth on the nanometre scale. However, there is a particular wave-like superstructure. The average width of the superstructure on the surface is approx. 500 nm. Also, these structures suffer from low stability. The dense sponge-like structure partly rips and sometimes delaminates at the silicon–sponge interface.

**Figure 8:**
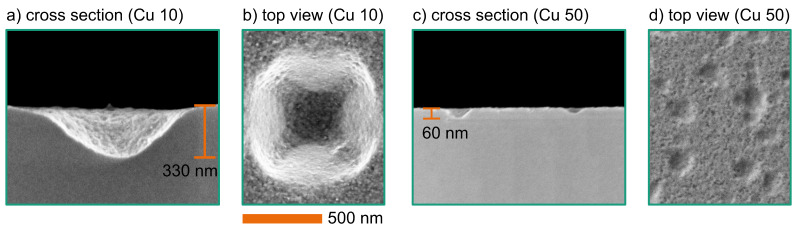
SEM images of etched structures using Cu particles, etched for 10 min with 1.73 mol/L HF and 10 mmol/L H_2_O_2_ ((a): cross section; (b): top view), and with 50 mmol/L H_2_O_2_ ((c): cross section; (d): top view).

Figure 8 shows SEM images of the etching results using Cu nanoparticles. The wafers were etched with 10 mmol/L of H_2_O_2_ (Figure 8a,b) or with 50 mmol/L of H_2_O_2_ (Figure 8c,d). The images clearly demonstrate that copper is a catalytic metal that supports the wet etching of silicon. However, copper dissolves in the harsh HF/H_2_O_2_ solution too fast for the etching of high aspect ratio templates. On the other side, this metal can be used to increase surface roughness. The complete dissolution of copper leaves a clean metal-free surface.

The wafer etched with the low H_2_O_2_ concentration shows large cavities after wet etching. Figure 8a and Figure 8b are centred at one of these cavities. The use of a higher H_2_O_2_ concentration leads to a faster copper dissolution. Therefore, the cavities in Figure 8c,d are significantly smaller.

**Figure 9:**
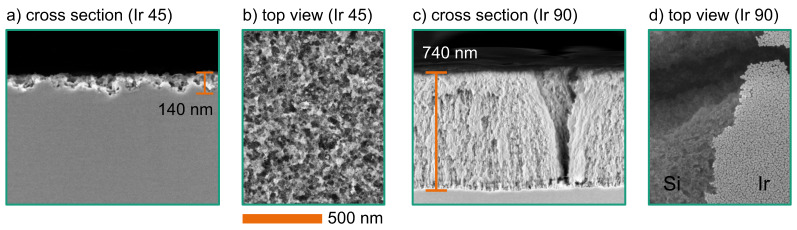
SEM images of etched structures (with 100 mmol/L H_2_O_2_ and 1.73 mol/L HF for 10 min) using Ir particles after 45 ALD cyles ((a): cross section; (b): top view), and after 90 ALD cycles ((c): cross section; (d): top view with partial Ir coverage).

In contrast to the previous experiments, the Ir samples were not integrated on the wafer-level. The Ir nanoparticles were deposited with ALD on top of rectangular silicon dies. These dies were not annealed for particle formation and had no additional PMMA masking layer. The Ir samples were handled and wet-etched as dies. Figure 9 shows the resulting structures after the silicon etching process. For both cases, the H_2_O_2_ concentration was 100 mmol/L. These results are representative for a wide range of H_2_O_2_ concentrations from 25 to 150 mmol/L. The particle density and distribution had a higher impact on the etching result than the H_2_O_2_ concentration.

The samples with the non-agglomerated Ir particles (after 45 ALD cycles, Figure 1e) are shown in Figure 9a,b. The etching results in randomly distributed porous structures. The mean etching depth of 140 nm is small compared to the results of Pd, Pt, or Au particles. However, these results demonstrate the feasibility of an iridium catalyst for the MACE process. Figure 9c,d shows the etching result of the dies with the partly agglomerated Ir particles. Here, the porous Ir film remains on top of the surface. This can be seen in Figure 9d where parts of the Ir film have been removed during sample preparation. The image shows the edge of the remaining Ir on the right side. Below this film, Si has formed a nanoporous structure. This structure reaches down to a depth of 740 nm. These structures also indicate that the MACE process with a perforated Ir film is dominated by the second etching mechanism with *n* = 2. However, this structure also suffers from low stability. The surface is affected by several fractures. This can be seen in Figure 9c,d.

### Comparison of reflectance measurements

**Figure 10:**
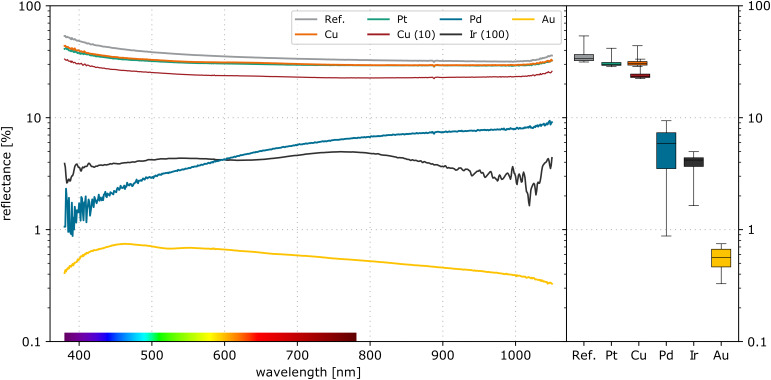
Reflectance of fabricated 3D templates, etched with 50 mmol/L H_2_O_2_ and 1.73 mol/L HF for 10 min. Additionally, the spectra of a Si reference, of a Cu wafer etched with 10 mmol/L H_2_O_2_, and of a Ir sample etched with 100 mmol/L H_2_O_2_ are given.

Figure 10 shows the measured reflectance of the fabricated high aspect ratio structures. The shown reflectance spectra are representative for processes with an H_2_O_2_ concentration of 50 mmol/L. In addition to that, the figure shows the result of a Cu-coated wafer etched with 10 mmol/L H_2_O_2_ and of a sample with Ir nanoparticles etched with 100 mmol/L H_2_O_2_. As reference the reflectance spectrum of an untreated silicon wafer is shown. The right side of the diagram summarises the reflectance spectra in boxplots.

The silicon reference shows an average reflectance of 35.5%. The dense surface of the Si structures obtained with Pt results in a high reflectance of 30.8%. This is mainly independent of the etching depth. Here, the homogeneous nanoporous Si film appears as a dense film with reduced refractive index. So the reflectance remains high. The rough surface of the Cu-coated wafers also shows a high reflectance. The wafer etched with 50 mmol/L H_2_O_2_ showed an average reflectance of 31.4%, while the low H_2_O_2_ concentration of 10 mmol/L resulted in a reduction to an average of 24.2%. The reflection behaviour is mainly related to scattering at the rough surface.

The samples with the Ir nanoparticles suffered from a strong local variation of the reflectance values. The shown measurement was carried out on the sample shown in Figure 9c,d. It has been etched with an H_2_O_2_ concentration of 100 mmol/L. The graph shows the potential of an integration using Ir nanoparticles. The mean reflectance can be decreased down to 4%. However, a substantial improvement in structure stability is required for reproducible results.

In contrast, the open nanowires obtained with Pd showed a significant reduction of the reflectance in a wide spectral range. For violet light, the measured reflectance was below 2%. In the near-infrared spectral range, the reflectance raised up to 9%. The average value over the measured range is 5.4%. For comparison, the measurement of the Si wafer with the Au particles is added to the graph. This wafer has been etched under the same etching conditions but shows a significantly better performance than the other wafers.

The reported reflectance values are summarised in Table 1. The average reflectance *R*_Mean_ represents the mean value of all measured data points without any weighting. The weighted reflectance *R*_Weight_ has been calculated according to [30] using normal direct solar irradiance.

**Table 1:** Summary of reported samples including catalyst material, H_2_O_2_ concentration, average reflectance (*R*_Mean_), and weighted average (*R*_Weight_).

catalyst	reference	Au	Au	Au	Au	Au	Cu	Cu	Pt	Pd	Ir (90)

*c*(H_2_O_2_) [mmol/L]	—	0	50	145	190	250	10	50	50	50	100
*R*_Mean_ [%]	35.5	34.0	0.56	0.76	0.22	0.22	24.2	31.4	30.8	5.4	4.0
*R*_Weight_ [%]	35.9	34.2	0.59	0.62	0.23	0.22	24.3	31.6	30.9	5.0	4.1

## Conclusion

Within this work, we could successfully demonstrate a wafer-level integration of high aspect ratio nanoporous templates. These self-assembled silicon nanostructures are locally defined. This is the basis of a local platform of high specific surface area for the fabrication of functional devices such as sensors, harvesters, or energy storage devices.

The selection of the noble metal and the noble metal particle formation process play an important role in the whole integration. The resulting structures range from a rough surface, over sponge-like nanoporous films to vertically aligned nanowires. Depending on the target application one can tune the geometry of the self-assembled structures in a wide range. This includes high aspect ratio silicon nanowires (so-called black silicon) with an average reflectance of less than 0.5%. The etching mechanism can also be optimised by the selection of the noble metal. The usage of gold will lead to straight structures, while etching with platinum-group metals (Pt, Pd, or Ir) yields predominantly nanoporous sponge-like structures.

We demonstrated the MACE process with the noble metals Au, Pt, Pd, Cu, and, as a novelty, with Ir. Depending on the contamination budget of the integrated system one can select an appropriate noble metal.

## Experimental

Here, a wafer-level integration scheme for the fabrication of high aspect ratio silicon templates is discussed. The whole process has been done on 150 mm p-doped (5–20 Ω·cm) silicon (100) wafers. The wafers have been coated with a 5 nm noble metal thin film using standard PVD methods. The deposition of gold and palladium was done by magnetron sputtering. The film thickness has been adjusted by the use of a calibrated deposition rate. Ion beam sputter deposition (IBSD) has been used to deposit platinum and copper. For copper deposition, the thickness has been controlled by an in situ micro quartz crystal sensor. The particle formation has been done by thermal annealing under vacuum conditions. The wafers with Cu and Pt have been annealed in vacuo, while the wafers with Au and Pd were annealed ex situ. Each annealing consists of a heating phase, 30 min of annealing, and a cool-down phase. The annealing temperatures were 300 °C for Au and Cu, 500 °C for Pd, and 590 °C for Pt, respectively.

The annealed wafers with the noble metal nanoparticles have been coated with a masking layer. We selected PMMA due to its chemical stability against HF and H_2_O_2_. These masking layers have been patterned with standard photo-resist and a proximity lithography step. The selected full wafer pattern consists of a series of open quadratic test fields. These were transferred from the photo-resist to the PMMA layer using a reactive ion etching process with oxygen.

In contrast to the wafer-level preparation, the iridium film was deposited by atomic layer deposition on rectangular Si(100) samples, using Ir(acac)_3_ and oxygen. Details are described by Genevée et al. [31]. The process was carried out with 45 and 90 cycles. One ALD cycle consists of pulsing Ir(acac)_3_ for 6 s, followed by purge of the reactor with inert gas for 60 s. The removal of the ligands is ensured by an O_2_ pulse for 2 s followed by a purge for 6 s.

Finally, the wafers (and Ir containing samples) have been wet-etched. This etching has been done in a single wafer bath with a total liquid volume of 800 mL. The etching bath consists of 730 mL of deionised water and 70 mL of HF (40 vol %). Additionally, a variable dose of H_2_O_2_ (30 wt %) has been added up to a volume of 20 mL. The etching solution had an HF concentration of 1.73 mol/L. The H_2_O_2_ concentration has been varied in a range of 0–0.24 mol/L. The typical concentration was 50 mmol/L. After an etching time of 10 min, the wafers have been rinsed with deionised water and dried with a spin rinse dryer or manually in an N_2_ stream.

A micro-spectrophotometer USPM-RU-W NIR (Olympus K. K.) was used to determine the reflectance. The measuring beam is focused on the sample surface yielding a small measuring spot with a diameter of about 70 µm under normal incidence of light. The reflectance was determined in the spectral range of 380–1050 nm using a BK7 and Si reference sample, respectively.

The detailed structure analysis has been carried out with a Zeiss Supra scanning electron microscope. The images have been acquired using the in-lens detector and an electron energy of 1.0 keV. The images with high material contrast have been acquired using the back-scattering detector and an electron energy of 10.0 keV. The particle analyses had been done on wafer-level. After wet etching of the silicon, the wafers were cut into small samples to get surface images as well as cross-sectional images.
